# Fusing Self-Attention and CoordConv to Improve the YOLOv5s Algorithm for Infrared Weak Target Detection

**DOI:** 10.3390/s23156755

**Published:** 2023-07-28

**Authors:** Xiangsuo Fan, Wentao Ding, Wenlin Qin, Dachuan Xiao, Lei Min, Haohao Yuan

**Affiliations:** 1 School of Automation, Guangxi University of Science and Technology, Liuzhou 545006, China; 100002085@gxust.edu.cn (X.F.); 221068323@stdmail.gxust.edu.cn (W.Q.); 221068407@stdmail.gxust.edu.cn (D.X.); 100000917@gxust.edu.cn (H.Y.); 2 Guangxi Collaborative Innovation Centre for Earthmoving Machinery, Guangxi University of Science and Technology, Liuzhou 545006, China; 3 Institute of Optics and Electronics Chinese Academy of Sciences, Chengdu 610209, China; minlei@ioe.ac.cn

**Keywords:** YOLOv5s, multi-head self-attention, CoordConv, NWD, target detection

## Abstract

Convolutional neural networks have achieved good results in target detection in many application scenarios, but convolutional neural networks still face great challenges when facing scenarios with small target sizes and complex background environments. To solve the problem of low accuracy of infrared weak target detection in complex scenes, and considering the real-time requirements of the detection task, we choose the YOLOv5s target detection algorithm for improvement. We add the Bottleneck Transformer structure and CoordConv to the network to optimize the model parameters and improve the performance of the detection network. Meanwhile, a two-dimensional Gaussian distribution is used to describe the importance of pixel points in the target frame, and the normalized Guassian Wasserstein distance (NWD) is used to measure the similarity between the prediction frame and the true frame to characterize the loss function of weak targets, which will help highlight the targets with flat positional deviation transformation and improve the detection accuracy. Finally, through experimental verification, compared with other mainstream detection algorithms, the improved algorithm in this paper significantly improves the target detection accuracy, with the mAP reaching 96.7 percent, which is 2.2 percentage points higher compared with Yolov5s.

## 1. Introduction

Infrared weak target detection is a key technology in the field of computer vision and is widely used in military and civilian applications, such as aerospace, precision guidance, infrared early warning, and drone detection [1]. At present, there are still many difficulties in infrared weak target detection technology. First, the target is far away from the IR detector, resulting in a small size for the target in the image, usually showing a few pixels, a low signal-to-noise ratio for the target, a relatively weak signal, and a lack of rich, detailed information [2]. In addition, long-distance imaging leads to a large image scene span and a complex background environment, which is easily affected by external factors such as weather and noise, resulting in the target being submerged in the background, which increases the difficulty of weak target detection. Due to the above difficulties, the existing weak target detection algorithms have limitations and have difficulty meeting practical needs. Therefore, it is important to study accurate and fast infrared weak target detection algorithms.

Traditional weak target detection algorithms are divided into single-frame detection and multi-frame detection algorithms, The single-frame detection algorithm detects weak targets within a single image frame, such as two-dimensional minimum mean square filtering [3], mathematical morphological methods [4], and local contrast metrics [5]. The single-frame detection algorithm is low in complexity, has less computation, and is easy to implement in hardware, but the anti-interference ability is poor, and it is difficult to achieve good detection results in complex environments [6]. Multi-frame detection uses the temporal and spatial information of multi-frame images combined with the motion trajectory of the target to perform target detection. Examples include the 3D matched filtering method [7], dynamic programming method [8], and particle filtering method [9]. The algorithm complexity of multi-frame detection is high, the amount of operations is very large, and the real-time performance in the detection process is poor, so it is used less in practical engineering.

In recent years, with the development of computer hardware and the maturation of artificial intelligence technology, more and more scholars have applied deep learning technology to weak target detection tasks. Convolutional neural networks rely on powerful feature extraction capabilities and have achieved excellent performance in target detection. Deep learning-based object detection algorithms are mainly divided into two-stage detection algorithms and one-stage detection algorithms [10]. Two-stage detection algorithms require the pre-generation of candidate boxes, which have high algorithm complexity and poor real-time performance. Common examples include Fast R-CNN [11] and Faster-RCNN [12]. One-stage detection algorithms do not require the generation of candidate boxes. They directly predict the category and position of the target through convolutional neural networks, achieving end-to-end real-time detection. Examples include SSD [13] and the YOLO series [14]. Deep learning-based detection algorithms have achieved excellent detection results in detecting larger targets. However, when the target becomes smaller, the detection accuracy of these algorithms still needs to be improved.

The YOLO algorithm was first proposed by Joseph Redmon in 2015, and its main idea is to consider the target detection task as a regression problem which can predict both the location and class of the target in the neural network. Thus far, the YOLO family of algorithms has been updated to the eighth generation, and with its fast detection speed and good detection accuracy, it has been applied to small target inspection by many scholars. Xu et al. proposed a shape distance clustering (SDC) model in small target ship detection to generate superior a priori frames and used lightweight cross-level modules (L-SCP) and network pruning to reduce model computation [15]. Hu et al. applied channel and spatial attention mechanisms in YOLOv4 to optimize feature representation in both the spatial and channel dimensions, improving the accuracy of ship detection. They also used a new loss function to improve training efficiency [16]. Kim et al. applied the efficient channel attention mechanism (ECA-Net) to YOLOv5 and proposed an efficient channel attention pyramid network, which achieved improved small object detection performance at a lower cost [17]. Ye et al. utilized high-resolution feature layers to utilize shallow details and location information and adopted a new feature fusion method to capture remote contextual information of small targets and suppress shallow noise interference, effectively improving the detection accuracy of small infrared targets [18]. Liu et al. introduced coordinate attention in YOLOv5, allowing the network to focus more on the position information of the target. They also added dilated convolution in the residual structure to expand the receptive field and extract more target features [19]. Zhou et al. proposed the YOLO-SASE detection algorithm, which takes super-resolution reconstructed images as input and combines a multi-level perceptual field structure and an attention mechanism. This method improves feature utilization [20]. Mou et al. improved the upsampling and downsampling modules of YOLOv5 using the STD module and the CARAFE operator, reducing feature loss during the scaling of images and achieving significant results [21]. Dai et al. improved the YOLOv5 loss function and the prediction frame filtering method while adding an attention mechanism to the network. This method improves the detection efficiency and accuracy of the algorithm [22].

The above algorithms have different improvements for small target detection and provide ideas for this paper’s research on infrared weak small target detection. To improve the accuracy of infrared weak target detection, this paper proposes an improved YOLOv5 weak target detection algorithm that fuses the transformer and coordinate convolution. The main contributions of this paper are as follows:

1. Introducing the Bottleneck Transformer module in the backbone section of YOLOv5s using a multi-head self-attention mechanism to enhance the global modeling capabilities of detection networks;
2. Adding CoordConv to the Neck section of YOLOv5s using the coordinate channel allows the convolution to perceive the coordinates to some extent during the learning process, improving detection accuracy;
3. Creating a two-dimensional Gaussian distribution in the target box to represent the importance of the pixel points using the normalized Gaussian Wasserstein distance instead of the CIOU as the similarity measure between the prediction frame and the true frame, effectively enhancing the weak target detection capability;
4. In this paper, the improved YOLOv5s algorithm is experimentally compared with the lightweight algorithms YOLOv3-tiny, YOLOv4-tiny, YOLOv4s, PP-YOLOEs, YOLOv7-tiny, the algorithm in this paper performs better in terms of detection accuracy, with an mAP reaching 0.967.

## 2. Materials and Methods

### 2.1. Yolov5 Target Detection Algorithm

**Figure 1:**
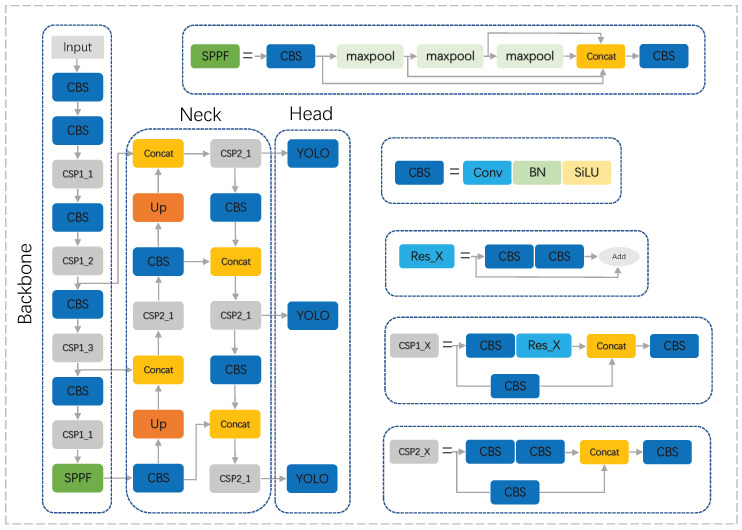
YOLOv5 network structure. X indicates that there are X identical stacks of residual blocks.

YOLOv5, as the latest phase of the target detection algorithm, has a fast detection speed and high recognition accuracy. Real-time detection can be achieved. The input image after a convolutional neural network’s forward propagation can directly predict the target bounding box and category. YOLOv5 is divided into four main sections: Input, Backbone, Neck, and Head. For the preprocessing of data in the input section of YOLOv5, the image data will first undergo Mosaic data enhancement, where several different images are stitched together according to random scaling, random cropping, and random alignment to increase the data sample, improving the algorithm’s robustness. At the same time, YOLOv5 will automatically calculate the anchor frame to match the target size. YOLOv5 uses CSPDarknet53 as the Backbone, which consists of the convolution, CSP residual structure, and SPPF for feature extraction. The Neck structure uses an FPN+PAN structure, with a top-down feature map of the FPN structure conveying strong semantic information and bottom-up transfer of the position characteristics from the PAN structure. The Neck fuses the feature maps of each level and obtains three feature maps of different sizes. The final output by the Head has the predicted information. The detection network structure of YOLOv5 is shown in Figure 1.

The loss function of YOLOv5 consists of three parts: classification loss, confidence loss, and localization loss. Among them, the binary cross-entropy loss is used for target confidence loss and classification loss, and the CIOU loss is used for the localization loss. The classification loss is used to calculate whether the anchor frame is accurate with the corresponding category, the confidence loss is used to calculate the confidence level of the network, and the localization loss is used to calculate the error between the predicted frame and the real frame. The loss function is shown in Equation (1):

(1) $$Loss=\lambda_{1}L_{cls}+\lambda_{2}L_{obj}+\lambda_{3}L_{\mathrm{CIOU}}$$

The CIOU loss takes into account the overlapping area of the two rectangular frames, the distance between the center points, and the aspect ratios of the two rectangular frames. In Equations (2)–(5), IOU denotes the ratio of the intersection area of rectangular boxes A and B to the merging area, ρ represents the distance between the center points of the rectangular box, *c* denotes the diagonal length of the outer rectangle of the two rectangular boxes, *v* denotes the similarity of the aspect ratios of two rectangular boxes, α is the impact factor, $w_{gt}$ and $h_{gt}$ indicate the width and height of the true frame, respectively, and $w_{p}$ and $h_{p}$ indicate the width and height of the prediction box, respectively. When the degree of overlap between the rectangular boxes is small, the smaller α is, the smaller the influence of *v* is in the loss function, and the optimization direction at this time is the distance between the rectangular boxes. When the overlap between the rectangular boxes is large, the larger α is, the greater the effect of *v*. The optimization direction is the width-to-height ratio between the rectangular boxes:

(2) $$IOU=\frac{A\cap B}{A\cup B}$$

(3) $$CIOU=IOU-\frac{\rho^{2}}{c^{2}}-\alpha v$$

(4) $$v=\frac{4}{\pi^{2}}{\left(\arctan\frac{w_{\mathrm{gt}}}{h_{\mathrm{gt}}}-\arctan\frac{w_{p}}{h_{p}}\right)}^{2}$$

(5) $$\alpha =\frac{v}{1-IOU+v}$$

Currently, YOLOv5’s authors offer five versions, depending on the needs of different tasks: YOLOv5n, YOLOv5s, YOLOv5m, YOLOv5l, and YOLOv5x. The depth and width of the network varies for each version. Smaller models have faster detection, and larger models have better detection performance. The infrared weak target detection task requires high detection accuracy in addition to the high real-time algorithm. The structure of YOLOv5s is simple, and the model size is only 14 M, while the detection is fast and does not reduce the accuracy too much compared with other algorithms, Therefore, YOLOv5s is more suitable for infrared weak target detection task needs.

### 2.2. Improve Yolov5s

**Figure 2:**
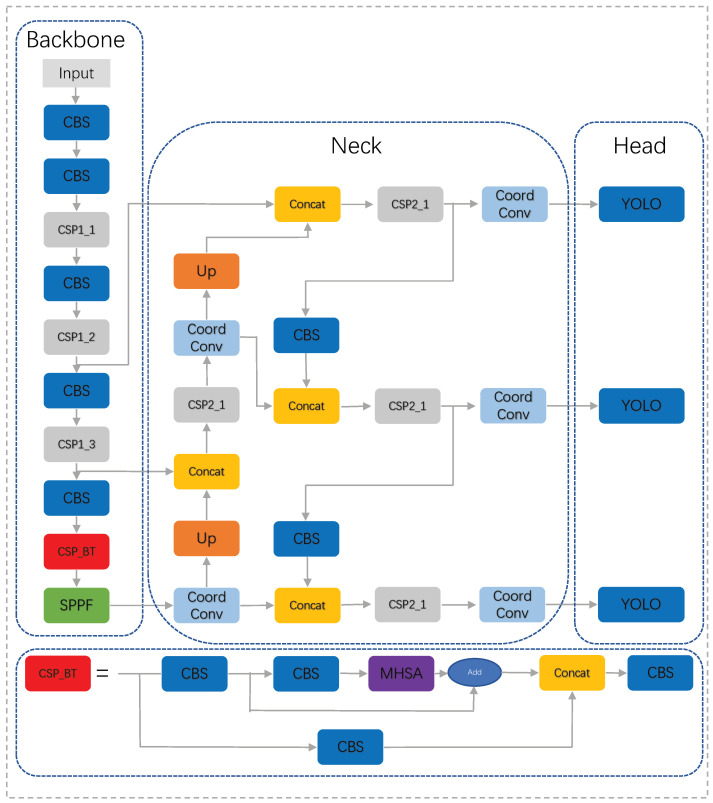
Improved YOLOv5s network structure. We introduced the CSP Bottleneck Transformer structure in the Backbone and added CoordConv to the Neck.

To improve the detection accuracy of infrared weak targets, we improve and optimize the YOLOv5s target detection algorithm. First, we add a Bottleneck Transformer to the CSP residual structure of the Backbone network. Second, CoordConv is added to the Neck structure, and CoordConv senses the position information by adding coordinate channels, obtaining more informative feature maps and improving model generalization. Finally, the CIOU loss function is changed to the normalized Guassian Wasserstein distance (NWD) loss to reduce the sensitivity to the weak target position’s bias transformation. The improved detection network is shown in Figure 2.

#### 2.2.1. Bottleneck Transformer

**Figure 3:**
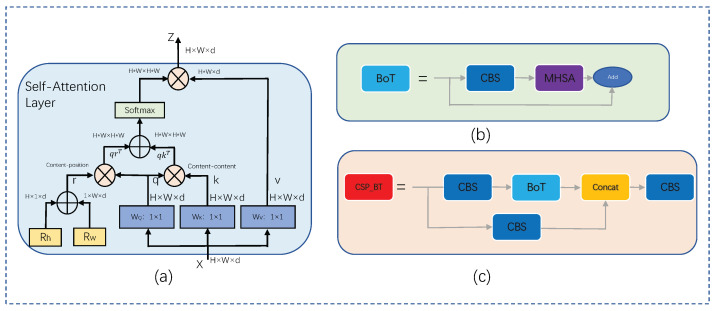
Design of Bottleneck Transformer. (**a**) Multi-head self-attention (MHSA) layer used in the Bottleneck Transformer. (**b**) Bottleneck Transformer structure. (**c**) Bottleneck Transformer with added CSP structure.

Ordinary convolution operations can effectively extract local feature information. However, in the task of target detection, global information is also very important. Using convolutional neural networks requires more layers, and global modeling can be achieved very easily and effectively using Transformer’s multi-head self-attention mechanism, improving the performance of target detection. We combine the convolutional neural network with the self-attention mechanism and utilize the self-attention mechanism to capture the global dependency, which makes up for the limitations of the convolutional network, and at the same time, the convolutional network can provide local spatial information for the self-attention mechanism, which enriches the representation. Since the computational amount of the self-attention mechanism is proportional to the size of the image, it will consume a lot of computational resources when processing high-resolution images. While the convolutional neural network obtains abstract and low-resolution feature maps after several downsampling operations, self-attention is inserted in the low-resolution feature maps, and the self-attention mechanism is utilized to process the information contained in the feature maps captured by the convolution. The last CSP residual module of YOLOv5 is a low-resolution feature map obtained after five downsamplings, replacing the 3 × 3 convolution in the residual network with a multi-head self-attention layer. We call this the Bottleneck Transformer [23] structure, which achieves global computation of feature maps without adding too much computation and improves the global modeling capability of the model. The structure of the multi-head self-attention layer is shown in Figure 3.

In the Figure 3, $R{}_{h}$ and $R_{w}$ are the relative position codes of the height and width, respectively, *q*, *k*, and *v* stand for query, key, and value, respectively, + and × denote element summation and matrix multiplication, respectively, and 1×1 means point-by-point convolution, with the input feature matrix having $W_{Q}$, $W_{K}$, and $W_{V}$. The dimension size is H×W×d. We initialized the height and width of the two position codes $R{}_{h}$ and $R_{w}$, respectively, and added the broadcast mechanism to obtain the position code *r*. The dimensions of the four parameters *q*, *k*, *v*, and *r* are H×W×d, *q* and *r* are multiplied by the matrix to obtain the content position output matrix, $qr^{T}$, *q*, and *k* are multiplied by the matrix to obtain the content position output matrix, and $qk^{T}$, $qr^{T}$, and $qk^{T}$ are matrix summed and Softmax normalized. We obtained an HW×HW size attention matrix. Finally, this was multiplied with the value projection *v* matrix to obtain the output value Z, where the output Z aggregates the global information. The computation of the multi-head self-attention layer was performed in parallel, and we used four heads. This can learn global dependencies from different representation subspaces, and the global information provided by self-attention can enhance the network’s semantic understanding of small targets as well as provide a global guide to the target location, which facilitates targeting of the target location.

#### 2.2.2. CoordConv

**Figure 4:**
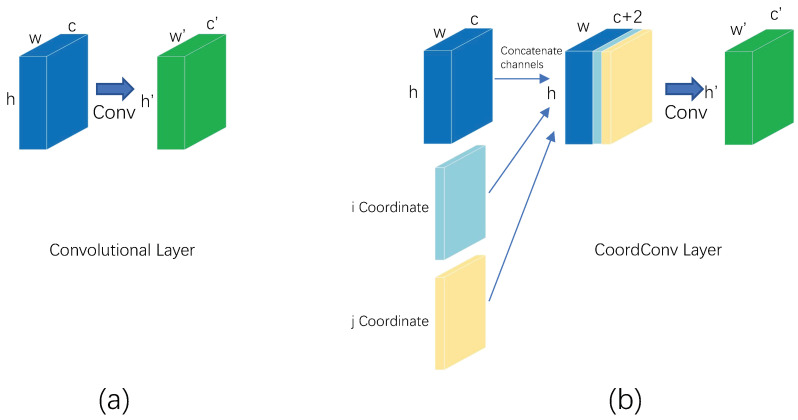
CoordConv structure diagram. (**a**) The calculation process of ordinary convolution. (**b**) The calculation process of CoordConv.

In convolutional neural networks, ordinary convolution learns spatially localized features with translational invariance, but it is unknown as to where in the image that information is located. In order to be able to sense the location information effectively, we added CoordConv [24] to the Neck structure of YOLOv5, whose structure is shown in Figure 4. Compared with normal convolution, CoordConv adds two coordinate channels to the input to represent the coordinate information of each pixel point: one for the x coordinate and one for the y coordinate. Splicing the two coordinate channels with the input channel followed by a convolution operation provides the network with spatial information, which helps the network to understand the spatial correspondence of the features in the image. With the spatial information, the network can build stronger spatial modeling capabilities, enhance the understanding of local and global locations, and improve location-based reasoning.

If CoordConv convolution learns to coordinate information, then CoordConv has certain translation dependence. If CoordConv does not learn to coordinate information, then coordinate convolution is equivalent to ordinary convolution, which retains the translation invariance of convolution, and thus CoordConv can choose whether to retain the translation invariance of traditional convolution according to different task requirements. In the infrared weak target detection task, the target is small, and the difference with the background is not obvious. Its coordinate position in the image is especially important. CoordConv takes the feature map with coordinate information as input, and the output gets the feature map, which contains the content information and coordinate spatial information. This can enhance the modeling of the weak target position information and enable the network to extract the generalized features which do not depend on the absolute position so as to improve the generalization ability of the model.

#### 2.2.3. Normalized Gaussian Wasserstein Distance

Although the CIOU considers the overlap area, centroid distance, and aspect ratio, the IOU-based metric is very sensitive to the position deviation of weak targets. Since targets occupy fewer pixel points, a slight position deviation will cause a sharp change in the IOU, which is not a problem for large-sized targets, and the CIOU is not the best method for weak target detection tasks. To solve this problem, we use the normalized Gaussian Wasserstein distance (NWD) method [25] to calculate the similarity between the predicted frame and the real frame. For a weak target which has a shape approximating a circle and whose size does not fill the entire wraparound box, there are other background pixels inside the wraparound box, and the target’s pixels are mainly concentrated close to the center of the wraparound box, with fewer target pixels at the boundaries. Therefore, the enclosing frame can be modeled as a two-dimensional Gaussian distribution to represent the importance of the distribution of target’s pixel points within the frame. Specifically, the coordinates of the center point of a bounding box are $\left(c_{x},c_{y}\right)$, the width is *w*, and the height is *h*. Then, the equation of the tangent ellipse of the bounding box can be expressed as follows:

(6) $$\frac{{\left(x-c_{x}\right)}^{2}}{{\left(\frac{w}{2}\right)}^{2}}+\frac{{\left(y-c_{y}\right)}^{2}}{{\left(\frac{h}{2}\right)}^{2}}=1$$

The probability density function of a two-dimensional Gaussian distribution can be expressed as follows:

(7) $$f\left(\left.X\right|\mu ,\Sigma \right)=\frac{\exp\left(-\frac{1}{2}{\left(X-\mu \right)}^{T}\Sigma^{-1}\left(X-\mu \right)\right)}{2\pi {\left|\Sigma \right|}^{\frac{1}{2}}}$$

where *X* denotes the coordinates $\left(x,y\right)$, μ denotes the mean vector, and Σ denotes the covariance matrix. When ${\left(x-\mu \right)}^{T}\Sigma^{-1}\left(x-\mu \right)=1$, the inner tangent ellipse of the bounding box is the density profile of the 2D Gaussian distribution. Thus, the bounding box $R=(c_{x},c_{y},w,h)$ can be modeled as a two-dimensional Gaussian distribution $N\left(\mu ,\Sigma \right)$. The formula can be expressed as follows:

(8) $$\mu =\left(\begin{array}{c} c_{x}\\ c_{y} \end{array}\right),\Sigma =\left(\begin{array}{cc} \frac{w^{2}}{4} & 0\\ 0 & \frac{h^{2}}{4} \end{array}\right)$$

Therefore, we used the distribution distance between two Gaussian distributions to represent the similarity between the predicted and real frames.

We used the Wasserstein distance from optimal transmission theory to compute the distribution distance of two two-dimensional Gaussian distributions. For two 2D Gaussian distributions $\mu_{1}\left(m_{1},\Sigma_{1}\right)$ and $\mu_{2}\left(m_{2},\Sigma_{2}\right)$, their Wasserstein distance can be defined as

(9) $$W_{2}^{2}\left(\mu_{1},\mu_{2}\right)={∥m_{1}-m_{2}∥}_{2}^{2}+{∥\Sigma_{1}^{\frac{1}{2}}-\Sigma_{2}^{\frac{1}{2}}∥}_{F}^{2}$$

For two bounding boxes $A=(c_{x1},c_{y1},w_{1},h_{1})$ and $B=(c_{x2},c_{y2},w_{2},h_{2})$ with Gaussian distributions $N_{1}$ and $N_{2}$, the Wassertein distance can be expressed as follows:

(10) $$W_{2}^{2}\left(N_{1},N_{2}\right)={∥\left({\left[c_{x1},c_{y1},\frac{w_{1}}{2},\frac{w_{1}}{2}\right]}^{T},{\left[c_{x2},c_{y2},\frac{w_{2}}{2},\frac{w_{2}}{2}\right]}^{T}\right)∥}_{2}^{2}$$

The distance metric is not used to represent the similarity, and thus it is normalized to represent the similarity metric in exponential form as shown in Equations (11) and (12). Compared with the IOU, the NWD-based method has a smoother positional deviation transformation for weak targets, which is more suitable for weak target detection:

(11) $$NWD\left(N_{a},N_{b}\right)=\exp\left(-\frac{\sqrt{W_{2}^{2}\left(N_{a},N_{b}\right)}}{C}\right)$$

(12) $$Loss_{NWD}=1-NWD\left(N_{p},N_{g}\right)$$

## 3. Results

### 3.1. Dataset

**Figure 5:**
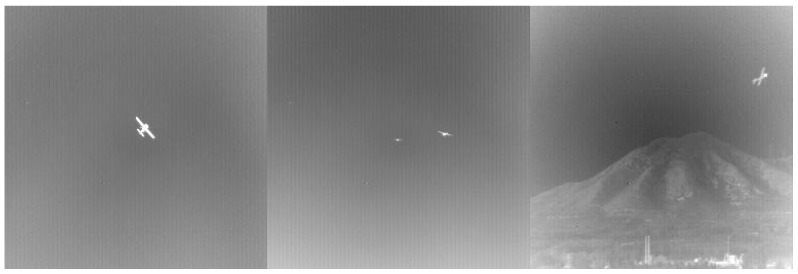
High signal-to-noise ratio image.

The datasets used in this study are the publicly available ground-to-air background infrared weak UAV dataset [26] and the anti-UAV anti-drone challenge competition dataset. There are 22 sequences of data in the dataset, and the image resolution size is 256 × 256. However, some of the images in this dataset have high signal-to-noise ratios, large target sizes in the images, rich details, clear edge contours, pure backgrounds, and no obvious noise, as shown in Figure 5, and the image continuity is too high, making them not suitable for weak target detection task requirements, Therefore, we eliminated high signal-to-noise ratio images while extracting one image every five images at intervals to ensure that the similarity between images was not too high. The video segments matching the weak target feature size and relatively low signal-to-noise ratio were selected from the anti-UAV anti-drone challenge data, and the videos were extracted into images every five frames and manually labeled using LabelImg software. Finally, 5358 pictures of different scenes were obtained as the dataset. The sample numbers of the datasets for different scenes are shown in Table 1.

**Figure 6:**
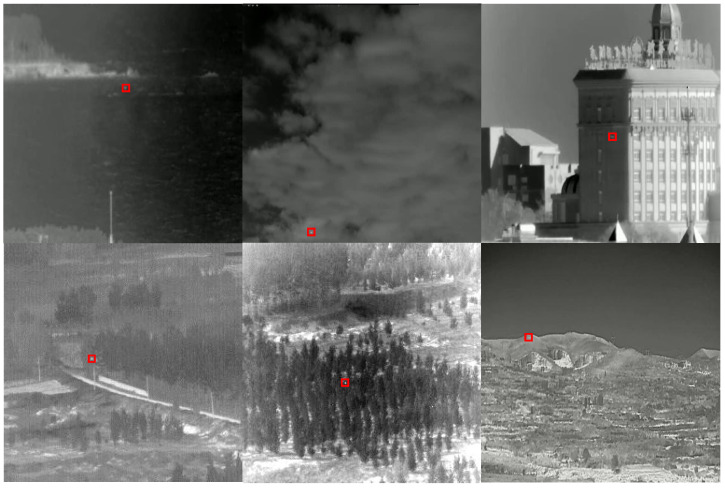
Sample dataset of six scenarios. The red boxes are real targets.

In this dataset, the data of weak targets with different backgrounds are shown in Figure 6. The data with the target background of sky and sea have a relatively high signal-to-noise ratio, less background interference, and a simple detection task, so fewer data samples were selected for this experiment. In the context of woodlands, mountains, roads, and buildings, there is more background interference, and the target signal-to-noise ratio is low, making detection relatively difficult. Thus, selecting more data samples for training could improve the robustness and generalization performance of the detection network.

### 3.2. Experimental Environment

In the training process, we divided the dataset into a training set, validation set, and test set at a ratio of 7:2:1. Since the preset anchor box size of YOLOv5 is based on the coco dataset, which does not meet the target size of this paper, we utilized the k-means++ algorithm to manually compute the size of the anchor box and obtain an anchor box suitable for the weak target data and sizes, as well as replace the anchor box of the original coco dataset. We used the development environment and training parameters shown in Table 2 and Table 3.

### 3.3. Evaluation Metrics

To better evaluate the performance of the improved YOLOv5s algorithm, this paper mainly uses the Precision, Recall, F1 score, and mean average precision (mAP) as the evaluation metrics of the algorithm. Finally, P-R curves were drawn to evaluate the algorithm’s performance.

The formula for Precision is

(13) $$Precision=\frac{TP}{TP+FP}$$

The precision rate indicates the percentage of samples identified as targets by the target detection algorithm that are actually targets. TP denotes true cases, or the number of samples predicted to be positive cases that are actually positive cases, and FP denotes false positive cases, or the number of samples predicted to be positive cases but are actually negative cases.

The formula for Recall is

(14) $$\mathrm{Recall}=\frac{TP}{TP+FN}$$

*Recall* indicates how many of the samples that are actually targeted are accurately detected. FN denotes the number of false negative cases, or the number of samples for which the predicted negative cases are actually positive cases.

The formula for the F1 score is

(15) $$F1=\frac{2\times Precision\times Recall}{Precision+Recall}=\frac{2TP}{2TP+FP+FN}$$

Precision and recall are generally negatively correlated; the higher the precision, the lower the recall. The F1 score can balance the impact of accuracy and recall and comprehensively evaluate the detection model. The higher the F1 score, the better the model performance.

The formula for calculating the average precision (AP) is

(16) $$mAP=AP=\int_{0}^{1}P\left(R\right)dR$$

The relationship between precision and recall can be expressed as a P−R curve. The area enclosed by the P−R curve and the coordinate axis is the average accuracy (AP) of the target. The AP represents the average accuracy of the algorithm for a certain class of targets, while the mAP represents the average of all classes of the AP. Since the detection target is a single class, the AP is equal to the mAP.

### 3.4. Analysis of Results

#### 3.4.1. Comparison of Different Algorithms

The weak target detection algorithm studied in this paper is mainly used in military and civilian applications, which require high real-time performance of the algorithm. Therefore, we used a one-stage detection algorithm to compare the performance with the improved algorithm in this paper under the same conditions. The first-stage detection algorithm is based on the YOLO series. The algorithm chosen for improvement in this paper was the smaller model of YOLOv5s, and the algorithm we chose for comparison was the lightweight detection network for each version of the YOLO series. Finally, we chose YOLOv3-tiny, YOLOv4-tiny, YOLOv4s, YOLOv5s, PP-YOLOEs, and YOLOv7-tiny with the same dataset for comparison with the algorithm in this paper. The results are shown in Table 4:

From the data in the table, it can be seen that YOLOv4-tiny detection was poor and far from the actual application requirements. The detection accuracy of YOLOv3-tiny and YOLOv7-tiny was lower compared with YOLOv5s, and the YOLOv3-tiny network structure was simpler and therefore faster. YOLOv4s is based on the network structure of YOLOv4, and it was realized by reducing the number of channels and the amount of residual structure stacking according to the design of YOLOv5s. Its detection results were slightly lower compared with YOLOv5s. YOLOv5s works best in IR weak target detection. PP-YOLOEs had a large gap between the precision and recall, resulting in poor mAP results as well as a low detection speed compared with other algorithms. Although YOLOv7 is an improved version of YOLOv5, the improved optimization of the YOLO algorithm is based on the coco dataset, and the latest version of the YOLO algorithm is not necessarily the most suitable one for the detection task in a particular scene. Overall, the improved YOLOv5s detection algorithm in this paper performed best in terms of precision, recall, mAP, and F1 scores, because the improved algorithm increased the number of parameters and computation, thus decreasing the detection speed.

#### 3.4.2. Performance Comparison

**Figure 7:**
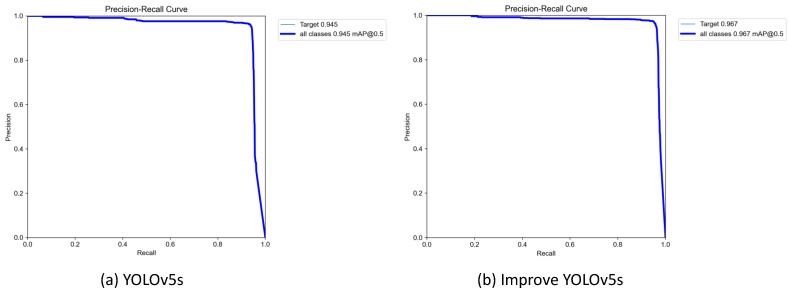
YOLOv5s P-R curve comparison.

We compared the performance of the YOLOv5s baseline algorithm with the improved YOLOv5s algorithm. The P-R curves were drawn separately after training using the same dataset, as shown in Figure 7. The P-R curve has the recall as the horizontal coordinate and precision as the vertical coordinate, and the area enclosed by the curve represents the mAP, which is used to evaluate the algorithm performance. It can be clearly seen in the figure that the improved YOLOv5s algorithm has a significantly larger area and a 2.2 percent improvement in its mAP compared with the P-R curve of the YOLOv5s baseline. It can be concluded that the improved YOLOv5s algorithm is the best for the detection of weak infrared targets, and the detection accuracy was significantly improved.

**Figure 8:**
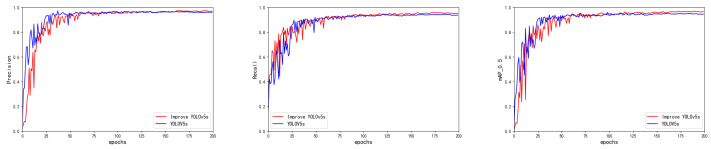
Precision, recall, and mAP comparison chart.

**Figure 9:**
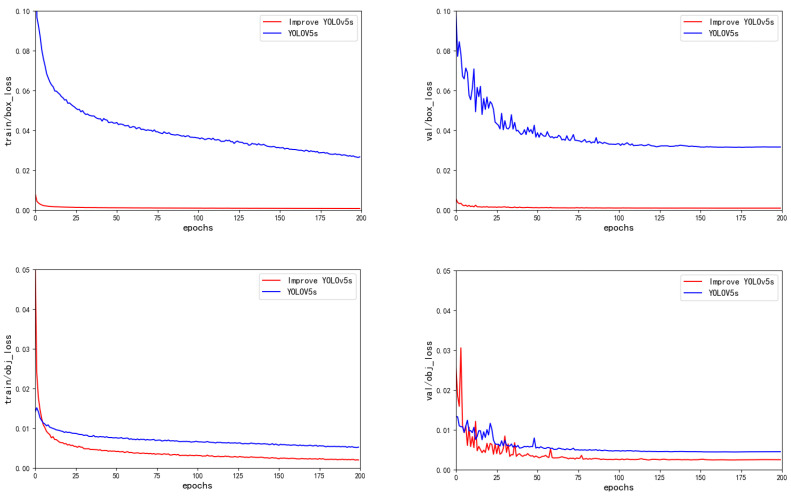
Comparison of loss curves.

We used TensorBoard to monitor the training data of the improved model during the training process, as shown in Figure 8. A total of 200 epochs was set for training, and the model performance metrics kept changing as the epochs increased. Among them, the mAP converged relatively slowly in the early stage. After 100 epochs, the curve leveled off. The improved YOLOv5s mAP was significantly higher than the YOLOv5s baseline network, and the precision and recall curves both improved. The graphs of the localization loss and confidence loss during training are shown in Figure 9. From the graphs, for both the training and validation sets, we can see that improved YOLOv5s achieved better results for both the confidence loss and localization loss, demonstrating the superiority of our improved algorithm.

#### 3.4.3. Comparison of Test Results

**Figure 10:**
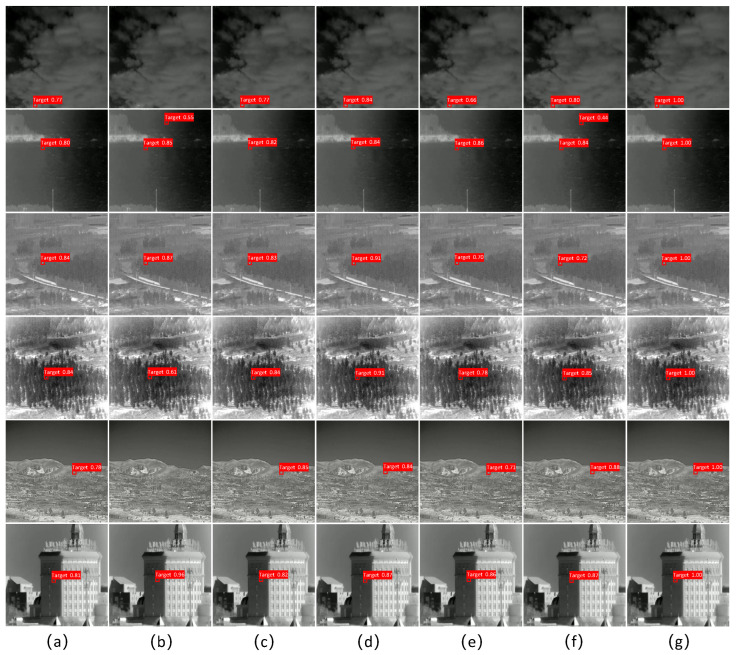
Comparison chart of detection results. (**a**) YOLOv3-tiny results. (**b**) YOLOv4-tiny results. (**c**) YOLOv4s results. (**d**) YOLOv5s results. (**e**) PP-YOLOEs results. (**f**) YOLOv7-tiny results. (**g**) Our method’s results.

We evaluated the detection performance of the YOLOv5s baseline and the improved YOLOv5s algorithm using data images from six different scenes: sky, mountain, water, building, road, and forest. We tested them using the same parameters, and the results are shown in Figure 10. YOLOv4-tiny and YOLOv7-tiny had false detections and missed detections. The detection recognition rate of the improved YOLOv5s algorithm was significantly better than those for other algorithms.

Analysis showed that adding a multi-head self-attention mechanism and CoordConv to the network structure of YOLOv5s can optimize the model parameters and make the detection model more expressive. The NWD loss avoided the problem of the CIOU’s sensitivity to position deviation of weak targets, which is more in line with the characteristics of weak targets and improves the recognition rate of weak targets. The experimental results show that our proposed method achieved good detection results.

In the testing process, although our improved YOLOv5s algorithm achieved good detection results while improving the detection accuracy, it also correspondingly reduced the detection speed by a small amount and increased the number of parameters of the model. The weak target detection approach of deep learning is based on being data-driven. Therefore, the limitation of the data volume in this study still leaves the generalization ability of the detection model inadequate, and YOLOv5 still has great room for improvement.

## 4. Conclusions

In summary, we studied how to improve the detection accuracy of infrared weak targets in complex scenes. In this paper, we first selected sample data that met the requirements of infrared weak target detection tasks in various complex scenes from two datasets and removed high signal-to-noise ratio images. We also used an interval multi-frame sampling method to reduce the continuity and similarity between data. Secondly, we improved and optimized the network structure of YOLOv5s by adding Bottleneck Transformer modules to the Backbone network of YOLOv5s, using the multi-head self-attention mechanism to improve the global modeling ability of the detection network and adding CoordConv to the Neck structure to perceive position information and improve the model’s generalization ability. At the same time, we replaced the CIOU loss function with the NWD loss. Finally, we compared the improved algorithm in this paper with YOLOv3-tiny, YOLOv4-tiny, YOLOv4s, YOLOv5s, PP-YOLOEs, YOLOv7-tiny, etc. From the evaluation index comparison table and P-R curve, we can see that the improved algorithm in this paper had the best performance, with the mAP reaching 96.7 percent. Overall, the research in this paper has improved the detection accuracy of infrared weak targets to a certain extent and achieved its research purpose.

## Figures and Tables

**Table 1:** Background distribution of the dataset.

Background	Woodland	Mountain	Sea	Sky	Roads	Architecture
Quantity	1351	1439	427	348	823	970

**Table 2:** Development environment.

Platform	Configuration
Integrated development environment	PyCharm
Scripting language	Python3.9
Operating system	Windows11
CPU	I5-12400F
GPU	NVIDIA GeForce RTX3060
Memory	16G
CUDA	11.7

**Table 3:** Training parameters.

Parameter	Configuration
Optimizer	SGD
Learning rate	0.01
Momentum	0.937
Decay	0.0005
Epochs	200
Batch size	32

**Table 4:** Comparison of algorithms.

Methods	Precision	Recall	mAP0.5	F1	FPS	Parameters	GFLOPs
YOLO3-tiny	0.948	0.912	0.93	0.93	204	8,666,692	12.9
YOLOv4-tiny	0.74	0.5	0.55	0.59	190	6,056,606	16.4
YOLOv4s	0.938	0.879	0.919	0.91	127	9,110,630	20.6
YOLOv5s	0.961	0.936	0.945	0.95	149	7,012,822	15.8
PP-YOLOEs	0.942	0.849	0.902	0.89	106	8,352,038	13.9
YOLOv7-tiny	0.941	0.86	0.905	0.9	163	6,007,596	13.0
Our Method	0.965	0.956	0.967	0.96	131	9,800,278	21.2

## Data Availability

The data used to support the results of this study are included in the article.

## Abbreviations

CSP	Cross-stage partial connection
NWD	Normalized Gaussian Wasserstein distance
MHSA	Multi-head self-attention
P-R	Precision-recall
mAP	Mean average precision
